# Combined Effects of Smoking and Alcohol on Metabolic Syndrome: The LifeLines Cohort Study

**DOI:** 10.1371/journal.pone.0096406

**Published:** 2014-04-29

**Authors:** Sandra N. Slagter, Jana V. van Vliet-Ostaptchouk, Judith M. Vonk, H. Marieke Boezen, Robin P. F. Dullaart, Anneke C. Muller. Kobold, Edith J. M. Feskens, André P. van Beek, Melanie M. van der Klauw, Bruce H.R. Wolffenbuttel

**Affiliations:** 1 Department of Endocrinology, University of Groningen, University Medical Center Groningen, Groningen, The Netherlands; 2 Department of Epidemiology, University of Groningen, University Medical Center Groningen, Groningen, The Netherlands; 3 Department of Laboratory Medicine, University of Groningen, University Medical Center Groningen, Groningen, The Netherlands; 4 Division of Human Nutrition, Wageningen University, Wageningen, The Netherlands; Bielefeld Evangelical Hospital, Germany

## Abstract

**Introduction:**

The development of metabolic syndrome (MetS) is influenced by environmental factors such as smoking and alcohol consumption. We determined the combined effects of smoking and alcohol on MetS and its individual components.

**Methods:**

64,046 participants aged 18–80 years from the LifeLines Cohort study were categorized into three body mass index (BMI) classes (BMI<25, normal weight; BMI 25–30, overweight; BMI≥30 kg/m^2^, obese). MetS was defined according to the revised criteria of the National Cholesterol Education Program’s Adult Treatment Panel III (NCEP ATP III). Within each BMI class and smoking subgroup (non-smoker, former smoker, <20 and ≥20 g tobacco/day), the cross-sectional association between alcohol and individual MetS components was tested using regression analysis.

**Results:**

Prevalence of MetS varied greatly between the different smoking-alcohol subgroups (1.7–71.1%). HDL cholesterol levels in all alcohol drinkers were higher than in non-drinkers (0.02 to 0.29 mmol/L, *P* values<0.001). HDL cholesterol levels were lower when they were also a former or current smoker (<20 and ≥20 g tobacco/day). Consumption of ≤1 drink/day indicated a trend towards lower triglyceride levels (non-significant). Concurrent use alcohol (>1 drink/day) and tobacco showed higher triglycerides levels. Up to 2 drinks/day was associated with a smaller waist circumference in overweight and obese individuals. Consumption of >2 drinks/day increased blood pressure, with the strongest associations found for heavy smokers. The overall metabolic profile of wine drinkers was better than that of non-drinkers or drinkers of beer or spirits/mixed drinks.

**Conclusion:**

Light alcohol consumption may moderate the negative associations of smoking with MetS. Our results suggest that the lifestyle advice that emphasizes smoking cessation and the restriction of alcohol consumption to a maximum of 1 drink/day, is a good approach to reduce the prevalence of MetS.

## Introduction

The metabolic syndrome (MetS) is present in approximately one-fourth of the adult European population [1] and mainly the result of overweight and obesity [2]. The syndrome is made up of a number of different components, namely high plasma glucose, high triglycerides, low high-density lipoprotein cholesterol, high blood pressure and enlarged waist circumference, which are all associated with excess adiposity. As a result of these metabolic abnormalities, there are four main health risks attributable to MetS, namely type 2 diabetes mellitus, cardiovascular disease, some types of cancer and all-cause mortality [2], [3]. The clinical management of MetS may depend on lifestyle changes and minimizing the components that characterize MetS. However, interventions aimed at weight loss and weight management showed only to be effective in the short term [4], [5]. By contrast, clinical and public health interventions were effective in reducing blood pressure and cholesterol in whole populations [6], [7]. Controlling the metabolic components might help to tackle the adverse effects of MetS resulting from the obesity epidemic. It is therefore important to investigate how lifestyle factors influence the components of MetS within people with a different body mass index (BMI), as a measure for obesity.

In this paper we focus on the two lifestyle factors smoking and alcohol consumption. Substantial evidence from epidemiological and clinical studies has shown that tobacco and alcohol are often used together, with smokers being more likely to drink than non-smokers and drinkers more likely to smoke than non-drinkers [8]. Although a dose-dependent association between tobacco use and the risk of developing MetS has been found [9], [10], the relationship between alcohol consumption and MetS is not consistent [11]–[18]. In addition to this, it is not well established how MetS is affected by the combination of these two lifestyle factors. The fact that tobacco and alcohol use do not affect the individual MetS components in a similar way makes the association complex. For instance, alcohol consumption is found to be positively correlated with high-density lipoprotein cholesterol (HDL-C) in a dose-dependent fashion, while smoking has the opposite effect [19]. Similarly, alcohol and smoking have opposite effects on insulin sensitivity, with alcohol having favorable effects [20], [21]. A further apparent contrast between these two factors is their effect on blood pressure. While alcohol consumption of three or more drinks per day increases blood pressure [22], the relationship between smoking and blood pressure is less clear or even non-existent [9], [23]. On the other hand, both smoking and alcohol consumption seem to have a positive association with triglyceride levels [9], [24] and abdominal obesity [9], [25], [26].

In an earlier paper, we reported on the relationship between smoking and MetS [9]. In the present study, we carefully assessed the combined effects of smoking and alcohol consumption on MetS and its individual components among normal weight, overweight and obese subjects from LifeLines, a very large population-based cohort study in the Netherlands (64,046 individuals). We also assessed whether the prevalence of MetS and its individual components was associated with the type of alcoholic beverage consumed. If we can identify how smoking and alcohol together influence MetS, we are better able to give tailored lifestyle advice to those at higher risk for developing MetS. To our knowledge, these lifestyle factors have not been explored directly or so extensively by other studies.

## Materials and Methods

### Study Design and Subjects

The LifeLines Cohort Study is a multidisciplinary prospective population-based cohort study with a unique three-generation design that examines the health and health-related behaviours of participants living in the north-eastern region of the Netherlands. More information about the LifeLines Cohort Study can be found elsewhere [27]. Similar to our previous paper [9] we included subjects of Western European origin (according to self-reported information in the questionnaire). They were aged between 18 and 80 years and participated in the study between December 2006 and December 2012. We excluded individuals who had missing data on BMI (n = 15), or on the variables needed to define MetS (n = 480), or whose questionnaires were incomplete with regard to smoking (n = 918) or alcohol consumption (n = 2,590). The current dataset comprised 64,046 individuals available for analyses. Before participating in the study, all participants provided written informed consent. The study protocol was approved conforming to the Declaration of Helsinki by the medical ethical review committee of the University Medical Center Groningen.

### Outcome Measures

#### Clinical measures

A fixed staff of well-trained technicians, who had a long experience in the clinical practice, used a standardized protocol to obtain blood pressure and anthropometric measurements: height, weight, and waist circumference. Systolic and diastolic blood pressures were measured 10 times during a period of 10 minutes, using an automated Dinamap Monitor (GE Healthcare, Freiburg, Germany). The size of the cuff was chosen according to the arm circumference. The average of the final three readings was used for each blood pressure parameter. Anthropometric measurements were measured without shoes. Body weight was measured to the nearest 0.1 kg. Height and waist circumference were measured to the nearest 0.5 cm. Height was measured with a stadiometer placing their heels against the rod and the head in Frankfort Plane position. Waist circumference was measured in standing position with a tape measure all around the body, at the level midway between the lower rib margin and the iliac crest.

#### Biochemical measures

Blood was collected in the fasting state, between 8.00 and 10.00 a.m., and transported to the LifeLines central laboratory facility at room temperature or at 4°C, depending on the sample requirements. On the day of collection, serum levels of total and HDL cholesterol were measured using an enzymatic colorimetric method, triglycerides using a colorimetric UV method, and LDL-C using an enzymatic method, all on a Roche Modular P chemistry analyzer (Roche, Basel, Switzerland). Fasting blood glucose was measured using a hexokinase method.

#### Definition of the Body Mass Index classes (BMI) and metabolic syndrome

Subjects were classified into three BMI classes: normal weight (BMI<25.0 kg/m^2^), overweight (BMI 25.0 to 30.0 kg/m^2^) or obese (BMI≥30.0 kg/m^2^), calculated as weight (kg) divided by height squared (m^2^). Metabolic syndrome was defined according to the revised criteria of the National Cholesterol Education Program’s Adult Treatment Panel III (NCEP ATP III) [28]. The NCEP ATP III stipulates the following five criteria for MetS: (1) systolic blood pressure≥130 mmHg and/or diastolic blood pressure≥85 mmHg and/or use of antihypertensive medication; (2) fasting blood glucose≥5.6 mmol/L and/or use of blood glucose-lowering medication and/or diagnosis of type 2 diabetes; (3) HDL cholesterol levels<1.03 mmol/L in men and<1.30 mmol/L in women and/or use of lipid-lowering medication; (4) triglyceride levels≥1.70 mmol/L and/or use of triglyceride-lowering medication; and (5) waist circumference ≥102 cm in men and≥88 cm in women. Individuals were diagnosed as having MetS if at least three of the five criteria were present. Medication use was self-reported. Diagnosis of diabetes mellitus was based either on self-report, or on the finding of a fasting plasma glucose>7.0 mmol/L.

### Data on Smoking, Alcohol Consumption and Medication Use

Information about smoking, alcohol consumption and medication use was collected from the self-administered questionnaires (http://www.p3gobservatory.org/catalogue.htm?questionnaireId=48). Non-smokers were those who had not smoked during the last month and had never smoked for longer than a year. Subjects were classified as a former smoker when they reported that they had smoked during a whole year, had not smoked during the last month and stopped smoking. Those who had smoked for longer than a year and had not stopped smoking were classified as current smoker. Total tobacco use of the current smokers was estimated by using the following quantities: 1 cigarette = 1 g tobacco, 1 cigarillo = 3 g tobacco and 1 cigar = 5 g tobacco. Moderate smoking was defined as 20 g/day or less, and heavy as more than 20 g/day [9].

Alcohol intake was based on the response to specific questions regarding intake frequency and the average number of units consumed on a drinking day. Individuals who reported not having consumed alcohol during the past month were considered non-drinkers. The number of alcoholic drinks per week was determined by multiplying the number of drinking days per week by the average number of units consumed on a drinking day. We then divided the number of alcoholic drinks/week by 7 in order to arrive at the number of alcoholic drinks per day. Individuals were classified into four groups according to their daily alcohol intake: 0 drinks/day (non-drinker), ≤1 drink/day (light drinker), >1 to 2 drinks/day (moderate drinker) and >2 drinks/day (heavy drinker). In the Netherlands a standard unit contains 9.9 grams of alcohol. For each type of alcoholic beverage, respondents indicated whether they consumed it never (0%), sometimes (30%), often (70%) or always (100%). We only included participants in the beer group, wine group (which included red wine, white wine, rosé, sherry, port, vermouth and madeira) or spirits/mixed drinks group (containing a spirit and a mixer) if that beverage type accounted for 70% or more of their alcohol consumption. Since very few participants consumed mainly spirits or mixed drinks, these two groups were pooled together.

All medications used by participants were classified according to the Anatomical Therapeutic Chemical (ATC) classification system. Medication use was then categorized into three groups: non users, ≤5 types of medication and >5 types of medication.

### Statistical Analysis

All analyses were conducted using IBM SPSS Statistics version 20 (IBM Corporation, Armonk, NY, USA). Continuous data are expressed as mean ± standard deviation (SD), and non-normally distributed data as geometric mean and interquartile range. For categorical variables, percentages are reported. Differences between the three BMI classes and four alcohol groups were tested using ANOVA for continuous data and chi-square test for categorical data.

Multivariate linear regression models were used to examine the associations between alcohol use, smoking and the five components of MetS, within the three BMI classes. Triglycerides and fasting blood glucose were log-transformed (natural log). Measured systolic and diastolic blood pressure were corrected for blood pressure-lowering medication by adding 10 mmHg and 5 mmHg, respectively. In Genomic Wide Association Studies this method is commonly used to approximate the true blood pressure values in treated subjects for high blood pressure [29]–[31]. This method is a better solution than ignoring treatment or excluding treated subjects [32], [33]. Analyses were stratified according to BMI class and smoking subgroups and adjusted for age (centered at the mean age of the total population (45y)), sex and the number of medications used. To assess beverage-specific associations with MetS and its components, we applied multivariate logistic regression models with non-drinkers as the reference group. MetS and the individual components were defined as ‘not meeting the criteria’ and ‘meeting the criteria’, as defined by the NCEP ATP III. These models were not stratified for BMI class and smoking subgroups due to the low number of drinkers who indicated consuming mainly beer, mainly wine or mainly spirits/mixed drinks. Models were adjusted for age, sex, BMI class, alcohol consumption subgroups, smoking subgroups and the number of medications used. To account for the number of independent tests, we applied a Bonferroni correction. Given the use of 12 independent tests (three BMI classes x four smoking subgroups), a P value of≤0.004 (0.05/12) was considered significant.

## Results

Overweight and obese subjects were slightly older than those with normal weight (table 1). Participants with a higher BMI had higher levels of systolic and diastolic blood pressure, serum triglycerides and blood glucose, and lower levels of HDL-C. The proportion of former smokers in the overweight and obese groups was higher than in the normal weight group, whereas the proportion of current smokers was approximately the same (normal weight 22.2%, overweight 20.8% and obese 19.4%). Among obese individuals, 25.8% were non-drinkers, while this percentage was much lower in overweight (15.0%) and normal weight (14.8%) individuals. Characteristics of the study population, according to alcohol consumption groups is available (Table S1).

**Table 1:** Characteristics of the total study population by BMI class.

Characteristics	BMI<25 kg/m^2^	BMI 25–30 kg/m^2^	BMI≥30 kg/m^2^	*P* value
n (%)	29,602 (46.2)	25,436 (39.7)	9,008 (14.1)	
**Age, yrs**	42±12	47±12	47±12	≤0.001
**Sex (m (%)/f)**	11,269 (38.1)/18,333	13,734 (54.0)/11,702	3,721 (41.3)/5,287	
**BMI, kg/m^2^**	22.6±1.7	27.1±1.4	33.4±3.4	≤0.001
**SBP, mmHg**	121±14	129±15	133±15	≤0.001
**DBP, mmHg**	71±8	76±9	77±9	≤0.001
**Total cholesterol, mmol/L**	4.9±1.0	5.2±1.0	5.2±1.0	≤0.001
**LDL-C, mmol/L**	3.02±0.86	3.39±0.90	3.38±0.90	≤0.001
**HDL-C, mmol/L**	1.59±0.40	1.40±0.36	1.28±0.33	≤0.001
**Triglycerides, mmol/L**	0.86 (0.63–1.11)	1.13 (0.79–1.55)	1.33 (0.93–1.83)	≤0.001
**Blood glucose, mmol/L**	4.78 (4.50–5.00)	5.04 (4.70–5.30)	5.31 (4.90–5.60)	≤0.001
**Waist circumference, cm**	82±8	94±8	107±10	≤0.001
***Smoking status***				
**Non-smoker, n (%)**	14,739 (49.8)	10,951 (43.1)	3,928 (43.6)	≤0.001
**Former smoker, n (%)**	8,282 (28.0)	9,179 (36.1)	3,332 (37.0)	≤0.001
**<20 gram tobacco/day, n (%)**	5,448 (18.4)	4,157 (16.3)	1,263 (14.0)	≤0.001
**≥20 gram tobacco/day, n (%)**	1,133 (3.8)	1,149 (4.5)	485 (5.4)	≤0.001
***Alcohol intake***				
**Non drinker**	4368 (14.8)	3,811 (15.0)	2,320 (25.8)	≤0.001
**≤1 drink/day**	15,933 (53.8)	12,420 (48.8)	4,220 (46.8)	≤0.001
**>1 to 2 drinks/day**	6,654 (22.5)	6,164 (24.2)	1,560 (17.3)	≤0.001
**>2 drinks/day**	2,647 (8.9)	3,041 (12.0)	908 (10.1)	≤0.001
***Medication use***				
**No medication, n (%)**	20,200 (68.2)	16,395 (64.5)	4,835 (53.7)	≤0.001
**≤5 types of medication, n (%)**	9,149 (30.9)	8,507 (33.4)	3,742 (41.5)	≤0.001
**>5 types of medication, n (%)**	253 (0.9)	534 (2.1)	431 (4.8)	≤0.001
**BP-lowering medication, n (%)**	1,145 (3.9)	2,450 (9.6)	1592 (17.7)	≤0.001
**Statin use, n (%)**	494 (1.7)	1,300 (5.1)	683 (7.6)	≤0.001
**TG-lowering medication, n (%)**	6 (0.1)	31 (0.1)	17 (0.2)	≤0.001
**Type 2 diabetes, n (%)**	99 (0.3)	302 (1.2)	339 (3.8)	≤0.001
**Oral anti-hyperglycaemic medication, n (%)**	67 (0.2)	233 (0.9)	274 (3.0)	≤0.001
**% fulfilling≥3 out of 5 MetS criteria**	792 (2.7)	4,492 (17.7)	4,388 (48.7)	≤0.001

Data are presented as mean ± SD, or geometric mean (interquartile range). Abbreviations: BMI = body mass index, SBP = systolic blood pressure, DBP = diastolic blood pressure, HDL-C = high density lipoprotein cholesterol, TG = triglycerides, BP = blood pressure, MetS = metabolic syndrome.

**Figure 1:**
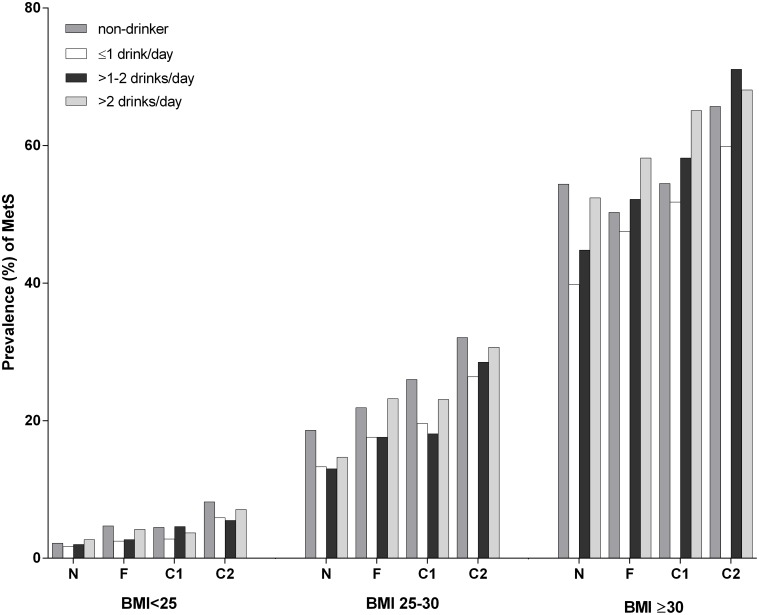
Prevalence of metabolic syndrome within the smoking and alcohol subgroups, according to BMI class. Top: BMI<25 kg/m^2^; middle: BMI 25–30 kg/m^2^; bottom: BMI≥30 kg/m^2^. BMI = body mass index N: non-smokers; F: former smokers; C1: smokers of <20 g tobacco/day; C2: smokers of ≥20 g tobacco/day.

For all three BMI classes, individuals were most frequently classified as being a non-smoker with an alcohol intake of≤1 drink/day (Table 2). The prevalence of MetS is given for each of the smoking and alcohol subgroups (Table S2 and figure 1). The percentages of subjects with MetS ranged widely across the different subgroups and BMI classes (normal weight: 1.7%–8.2%, overweight: 13.0%–32.1%, obese: 39.8%–71.1%).

**Table 2:** Distribution of the study population across the smoking and alcohol subgroups, according to BMI class.

	Non-smoker	Former smoker	Moderate smoker	Heavy smoker
BMI <25 kg/m^2^	(n = 14,739)	(n = 8,282)	(n = 5,448)	(n = 1,133)
	All, n (%)	All, n (%)	All, n (%)	All, n (%)
Non-drinker	2791 (18.9)	864 (10.4)	554 (10.2)	159 (14.0)
≤1 drink/day	8823 (59.9)	4364 (52.7)	2373 (43.6)	373 (32.9)
>1 to 2 drinks/day	2426 (16.5)	2299 (27.8)	1636 (30.0)	293 (25.9)
>2 drinks/day	699 (4.7)	755 (9.1)	885 (16.2)	308 (27.2)
	**Non-smoker**	**Former smoker**	**Moderate smoker**	**Heavy smoker**
**BMI 25–30 kg/m^2^**	**(n = 10,952)**	**(n = 9,179)**	**(n = 5,157)**	**(n = 1,149)**
	**All, n (%)**	**All, n (%)**	**All, n (%)**	**All, n (%)**
Non-drinker	2190 (20.0)	1026 (11.2)	458 (11.0)	137 (11.9)
≤1 drink/day	5980 (54.6)	4320 (47.1)	1737 (41.8)	383 (33.3)
>1 to 2 drinks/day	2025 (18.5)	2644 (28.8)	1218 (29.3)	277 (24.1)
>2 drinks/day	756 (6.9)	1189 (13.0)	744 (17.9)	352 (30.6)
	**Non-smoker**	**Former smoker**	**Moderate smoker**	**Heavy smoker**
**BMI≥30 kg/m^2^**	**(n = 3,928)**	**(n = 3,332)**	**(n = 1,263)**	**(n = 485)**
	**All, n (%)**	**All, n (%)**	**All, n (%)**	**All, n (%)**
Non-drinker	1319 (33.6)	658 (19.7)	235 (18.6)	108 (22.3)
≤1 drink/day	1907 (48.5)	1607 (48.2)	539 (42.7)	167 (34.4)
>1 to 2 drinks/day	475 (12.1)	694 (20.8)	294 (23.3)	97 (20.0)
>2 drinks/day	227 (5.8)	373 (11.2)	195 (15.4)	113 (23.3)

**Figure 2:**
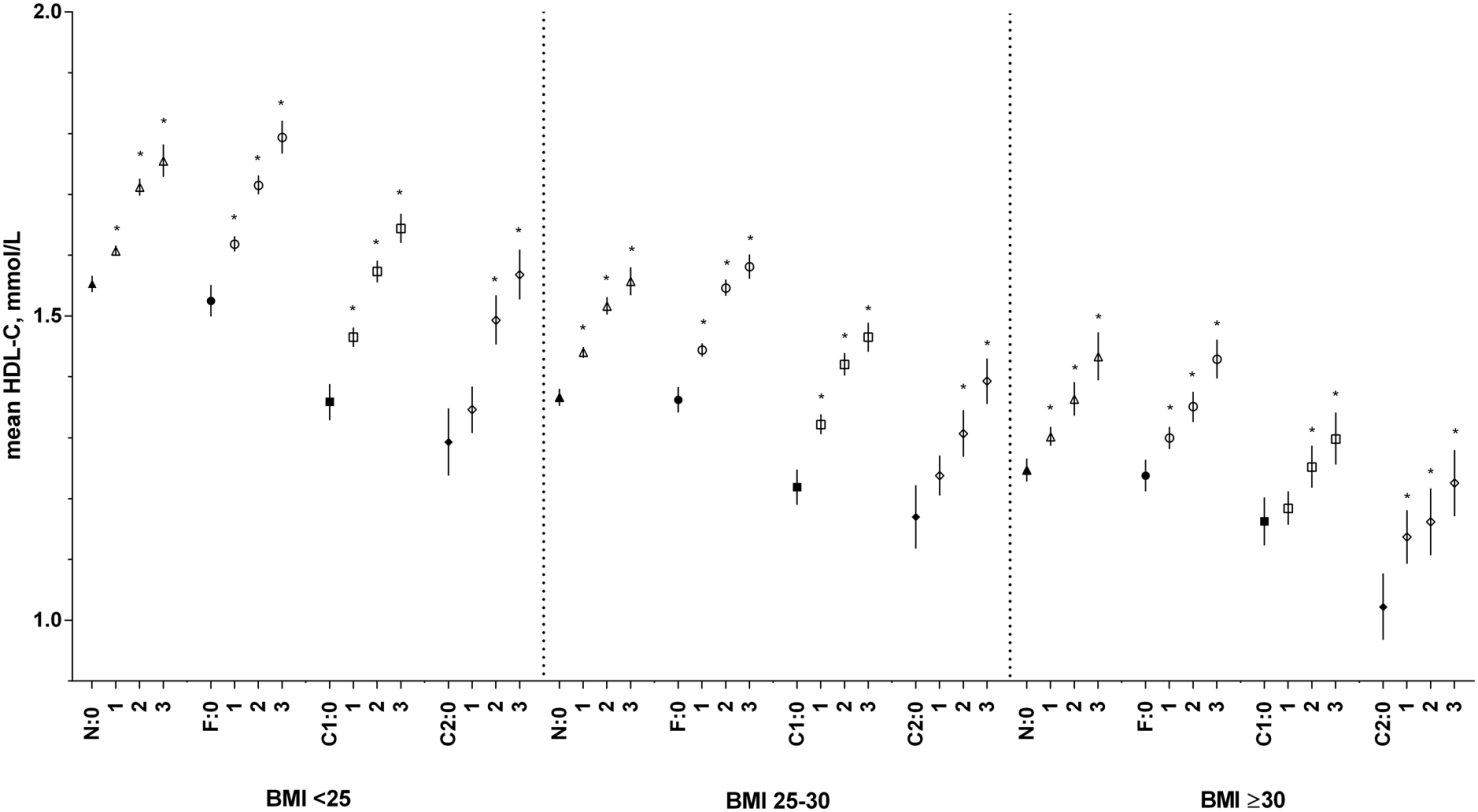
Results of the associations between the smoking-alcohol subgroups and HDL-C, according to BMI class. Adjusted for age (centered at the mean age of the total population (45y)), sex and the number of medications used. * indicates a significant difference within each smoking subgroup relative to the reference group of non-drinkers (shaded shape); P value≤0.001. N: non-smokers; F: former smokers; C1: smokers of <20 g tobacco/day; C2: smokers of ≥20 g tobacco/day. 0: non-drinker; 1: ≤1 drink/day; 2: >1–2 drinks/day; 3: >2 drinks/day. BMI = body mass index HDL-C = high-density lipoprotein cholesterol.

There was a dose-dependent increase in HDL-C levels with increasing levels of alcohol consumption, in all three BMI classes (*P* values ≤0.001) (Figure 2). When we looked at smoking status, we found that smokers had lower HDL-C levels than non-smokers, which decreased with the amount of tobacco used.

In all BMI classes, alcohol consumption of >1 drink/day showed a positive association with triglyceride levels (Figure S1a). Triglyceride levels also increased within each smoking subgroup. It should however be noted that only a few results reached statistical significance.

Although alcohol consumption does appear to increase fasting glucose levels, these differences were rather small and not statistically significant (Figure S1b). The relation between alcohol consumption and systolic blood pressure (SBP) and diastolic blood pressure (DBP) showed a J-shaped curve (Figure S1c and S1d). With higher blood pressure levels found among non-drinkers and moderate to heavy drinkers relative to light drinkers. Alcohol consumption of more than 2 drinks/day significantly increased systolic and diastolic blood pressure in normal weight and overweight individuals, in all smoking subgroups. The strongest association was within the group of heavy smokers, with increased blood pressures relative to non-drinkers by 6.1/3.1 mmHg (SBP/DBP) in normal weight individuals (*P*<0.001) and by 4.3/2.2 mmHg (*P* = 0.004) in overweight. The relationship between alcohol consumption and blood pressure was not significant in obese individuals.

Within normal weight individuals, higher amounts of alcohol consumption were associated with a larger waist circumference (Figure S1e). Among overweight individuals, alcohol consumption up to 2 drinks/day was associated with a slightly smaller waist circumference in non-smokers and former smokers compared to the non-drinkers within the same smoking class (all *P*≤0.001). A stronger association was found for obese individuals, showing a smaller waist up to 2.83 cm in non-smokers and up to 2.37 cm in former smokers (all *P*<0.001). Obese heavy smokers with an alcohol consumption of 1 to 2 drinks/day even had a 4.13 cm lower waist (*P* = 0.004).

**Figure 3:**
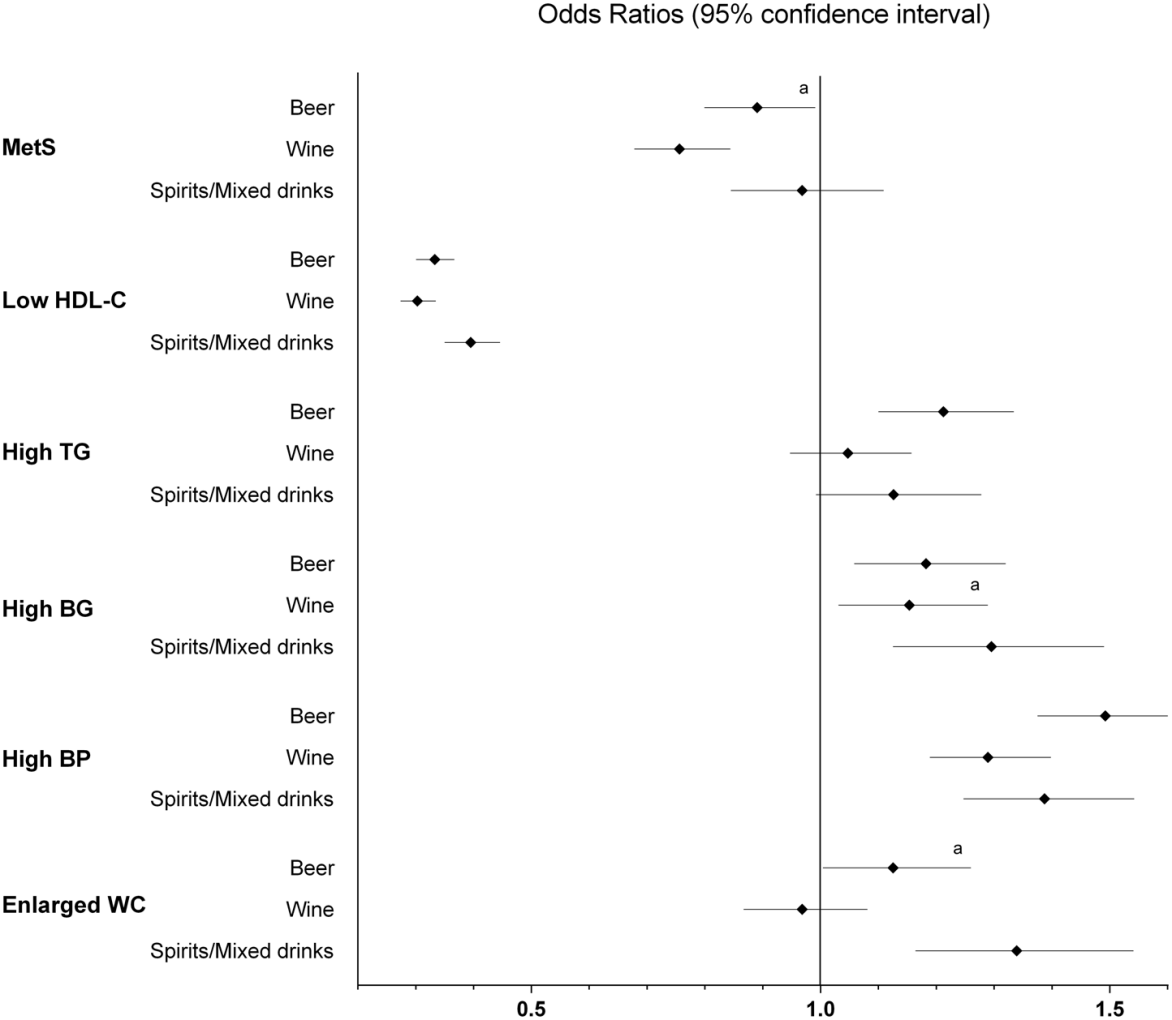
Odds ratios for MetS and the individual components according to type of alcoholic beverage. This analysis comprised 10,499 non-drinkers (reference group), 18,581 wine consumers, 20,894 beer consumers and 4,079 spirits/mixed drinks consumers, for all levels of alcohol consumption. Adjusted for age, sex, level of alcohol consumption, body mass index class, smoking subgroup and the number of medications used. Odds ratios were significant different from the reference group of non-drinkers at P value≤0.004. ^a^ indicates a significant difference relative to the reference group of non-drinkers at P value≤0.05. BG = fasting blood glucose; BP = blood pressure; HDL-C = high-density lipoprotein cholesterol; MetS = metabolic syndrome; TG = triglycerides; WC = waist circumference.

Table 3 summarizes the relationship of light, moderate or heavy alcohol consumption (relative to no alcohol consumption) and smoking on the individual MetS risk components. The analyses on the beverage-specific associations with MetS and its components (figure 3 **)**, showed that the odds ratio of having MetS was lower for wine drinkers than for non-drinkers (adjusted OR: 0.72; 95% CI: 0.68–0.84; *P*<0.001). Drinkers of all types of alcoholic beverages had a lower odds ratio of meeting the HDL-C criteria (all *P*<0.001) and a higher odds ratio of meeting those for hypertension (all *P*<0.001). Wine drinking did not affect the odds ratio for increased triglycerides, enlarged waist circumference or high fasting blood glucose (*P*>0.004).

**Table 3:** Overview of the relationships of light, moderate or heavy alcohol consumption (relative to non-consumption) and smoking on the individual MetS risk components.

Risk component	Light alcohol use	Moderate alcohol use	Heavy alcohol use	Smoking
HDL-cholesterol	↑↑	↑↑	↑↑	↓↓
Triglycerides	↓	↑	↑	↑
Blood glucose	N	N	↑	N
Blood pressure	↓	N	↑↑	N
Waist circumference	↕^a^	↕^a^	↕^a^	↑

Larger arrows and two arrows indicate a stronger association. N = neutral association.

a association depends on the body mass index: a larger waist circumference for BMI <25 kg/m^2^ and a smaller waist circumference for BMI≥25 kg/m^2^.

## Discussion

In this large population-based cohort study of the metabolic syndrome (MetS) among normal weight, overweight and obese subjects, we found smoking and light alcohol consumption to have opposing associations with MetS. In all BMI classes light alcohol consumption was associated with lower prevalence of MetS, explained by its favorable effects on HDL-C, triglycerides and waist circumference (only in overweight and obese individuals). Heavy alcohol consumption had unfavorable associations with individual MetS components. When compared with non-consumption of alcohol, we found wine consumption to be associated with a lower prevalence of MetS and the separate MetS components.

### Alcohol Consumption, Smoking and MetS

As might be expected, we found a wide range of MetS prevalence across the different smoking and alcohol subgroups and across the different BMI classes. Normal weight and overweight subjects with a light to moderate alcohol consumption had a lower prevalence of MetS, while for obese subjects this was the case for zero and light alcohol consumption. Compared to non-smokers, former, moderate and heavy smokers had a higher prevalence of MetS, regardless of the amount of alcohol consumed.

While our study shows a possibly protective association for alcohol in some cases, the literature reports conflicting results on the relationship between alcohol consumption and the prevalence of MetS. Finding no association [15], [16] or associations in different directions [11]–[14], [17], [18]. The small sample size of some of these studies and the fact that they did not take into account the smoking status of the participants, might explain the discrepancy in these results. However, we reported the prevalence of MetS in a large study population and stratified by smoking and alcohol subgroups, which gives a more reliable estimation.

### Effects of Smoking and Alcohol on Metabolic Risk Factors

We have previously reported the finding that former and current smokers have lower HDL-C levels and that this relationship is dose-dependent [9]. We now found that this negative influence of smoking on HDL-C may be suppressed by the favorable association between alcohol consumption and HDL-C. Here, we showed a dose-dependent association between alcohol consumption and higher levels of HDL-C, which is consistent with earlier studies [34]–[36]. The magnitude of the effects of alcohol consumption on HDL-C varied between 0.02 and 0.29 mmol/L. This means that in current smokers, moderate alcohol consumption is associated with similar mean HDL-C levels to those of their non-smoking and non-drinking counterparts within the same BMI class.

With regard to triglyceride levels, a cross-sectional population study has reported a U-shaped association between alcohol and triglycerides, with triglyceride levels the lowest in people with an alcohol consumption of 4 to 30 g/day [24]. Although our results revealed only a few significant associations for alcohol consumption, we did show higher triglyceride levels among former and current smokers, especially among those who drink more than 2 alcoholic drinks per day. One cross-sectional study in 3311 subjects from a Chinese population concluded that the effect of alcohol consumption on triglycerides was substantially greater for smokers of >20 cigarettes, than for lighter smokers and non-smokers [37]. In our population this was only true for the normal weight individuals. For overweight and obese individuals we cannot confirm these earlier findings (Figure S1a).

A dose-dependent relationship between alcohol consumption and risk of hypertension has recently been suggested in a large meta-analysis [22]. In the current study, alcohol consumption showed a ‘J-shaped’ relationship with systolic and diastolic blood pressure, within each BMI class.

Alcohol intake has been found to be highly correlated with both abdominal obesity [26] and increased risk for obesity [38], [39]. However, a prospective cohort study conducted among US men over a period of nine years, found no significant associations between changes in total alcohol consumption and gain in waist circumference [40]. In our study, we found that normal weight individuals who consumed alcohol had a larger waist circumference. In contrast, among overweight and obese individuals, light and moderate drinking was associated with a smaller waist than non-drinking. The biological mechanism by which alcohol consumption may reduce the waist circumference of overweight and obese individuals remains unclear. More studies are needed to confirm the differences that we observed between the three BMI classes.

We showed with our study that all metabolic parameters worsen with higher BMI. Reducing body weight would therefore be by far the best approach to reduce the prevalence of MetS. However, effective long-term successes of weight loss interventions are still missing [4], [5]. Recently, a paper has been published on the effects of metabolic mediators on coronary heart disease (CHD) and stroke within overweight and obesity [41]. They have estimated that nearly half of the excess risk for CHD and three-quarters of excess risk for stroke due to overweight and obesity were mediated through blood pressure, cholesterol and glucose. Blood pressure accounted for the highest percentage of excess risk for CHD (one-third) and stroke (two-third). Interventions that control metabolic factors might address a substantial proportion of the effect of high BMI on cardiovascular disease. However, to achieve full benefits from the interventions, reduction of body weight is recommended.

### The Effect of Beverage Type on MetS and its Components

The odds ratios of having MetS were lower for consumers of all types of alcoholic beverage than for non-drinkers, a finding also reported by Djoussé *et al.* [42]. However, in the present study, wine consumption resulted in the lowest odds ratio of having MetS and was the only significant association (*P*≤0.004). This suggests that the lowest odds ratio we observed for the wine drinkers, may be explained by other components than ethanol and/or the healthier lifestyle behavior associated with wine consumption [42], [43]. The overall metabolic profile of wine consumers was better than that of individuals who preferred other alcoholic beverages. Wine drinkers were also less likely to be current smokers (data not shown).

When we investigated the individual components of MetS we found the odds ratio of having low HDL-C levels to be lower for all beverage types than for non-drinkers. For beer consumption the only association found was a slightly higher odds ratio of having hypertriglyceridemia. The fact that the odds ratio was higher for beer consumers can be explained by the high carbohydrate content of beer, which is a well-known risk factor for increased triglycerides [44]. The finding that the odds ratio of having hypertension was lower for wine drinkers than for consumers of the other types of beverages, is also reported by another study [45]. Higher odds ratios for abdominal obesity were found for drinkers of spirits/mixed drinks and beer, although not significant for the latter (*P* = 0.043). These findings are in line with those reported by Valdstrup and colleagues [46].

### Strengths and Limitations

A major strength of our study is the nature of the study population, which is derived from the general population and both large and well characterized. We are the first to report on associations between the concurrent use of tobacco and alcohol and the various components of MetS. The sample size of 64,046 individuals allowed us to perform subgroup analyses within different smoking subgroups and BMI classes. We were even able to examine whether the presence of MetS and its components was associated with the type of alcoholic beverage consumed.

However, the study still has some limitations. Firstly, we were unable to make a distinction between abstainers and former drinkers. In this respect, the ‘J-shaped’ relationships found between alcohol consumption and both blood pressure and triglycerides might be explained by the lower health status of the non-drinking group (more medication users and type 2 diabetes patients) and possible inclusion of former drinkers. However, the ‘J-shaped’ relationship remained after exclusion of individuals with medication use. Secondly, the relationship between smoking, alcohol and MetS may be confounded by levels of physical activity and food intake. Smokers are known to be less physical active and have a less healthy diet than non-smokers [47]. Light and moderate alcohol consumption, in particular wine, is usually associated with a healthier lifestyle [47]. This notion is supported by the fact that beer and wine (which have the same ethanol content) showed different associations with MetS and its components. Such differences may be explained by lifestyle-related risk factors in consumers of beer, wine and spirits/mixed drinks that we could not control. Although we were not able to account for multiple critical lifestyle factors, we are the first to report in detail the combined effect of smoking and alcohol consumption on MetS. Thirdly, our findings could not support causality, due to the cross-sectional design of this study. A final point, which might be seen as a limitation, is the possibility of misclassification, since smoking and alcohol consumption was based on self-administered questionnaires. However, earlier studies showed that self-reported smoking status, tobacco use and alcohol consumption in the general population, can be used with notable confidence and provide an accurate estimation of the actual substance use [48]–[50].

## Conclusion

In our previous study we already showed that smoking was associated with a higher risk for MetS, explained by its negative influence on HDL-C, triglycerides and to a lesser extend waist circumference. With the current study we assessed the combined effect of smoking and alcohol consumption on MetS. In this large population-based cohort study we found that especially light alcohol consumption was associated with a favorable effect on the individual MetS components. Light alcohol consumption might therefore moderate the negative associations of smoking on MetS. Our results suggest that the lifestyle advice that emphasizes smoking cessation and the restriction of alcohol consumption to a maximum of 1 drink/day, is a good approach to reduce the prevalence of MetS. Maintaining a healthy body weight is recommended to fully benefit from this approach. These lifestyle advices may also help to prevent the onset of cardiovascular disease, since it is the main health risk attributable to MetS.

## Supporting Information

Figure S1a–e **Results of the associations between the smoking-alcohol subgroups and components of MetS, according to BMI class.** Adjusted for age (centered at the mean age of the total population (45y)), sex and the number of medications used. * indicates a significant difference within each smoking subgroup relative to the reference group of non-drinkers (shaded shape); P value≤0.001. ** indicates a significant difference within each smoking subgroup relative to the reference group of non-drinkers (shaded shape); P value≤0.004. N: non-smokers; F: former smokers; C1: smokers of <20 g tobacco/day; C2: smokers of ≥20 g tobacco/day. 0: non-drinker; 1: ≤1 drink/day; 2: >1–2 drinks/day; 3: >2 drinks/day. BMI = body mass index; BG = fasting blood glucose; DBP = diastolic blood pressure; SBP = systolic blood pressure; TG = triglycerides; WC = waist circumference.
(DOCX)

Table S1 **Characteristics of the total study population by alcohol subgroup.**
(DOCX)

Table S2 **Distribution of the study population across the smoking and alcohol subgroups, according to BMI class.**
(DOCX)
