# Correction: Combined Effects of Smoking and Alcohol on Metabolic Syndrome: The LifeLines Cohort Study

**DOI:** 10.1371/journal.pone.0105157

**Published:** 2014-08-05

**Authors:** The PLOS ONE Staff

The fourth author’s name is spelled incorrectly. The correct name is: H. Marike Boezen.
