# Supplementary material for: Combined Effects of Smoking and Alcohol on Metabolic Syndrome: The LifeLines Cohort Study
Source: PLoS One. 2014 Apr 29;9(4):e96406. doi: 10.1371/journal.pone.0096406 (PMC4004580; doi:10.1371/journal.pone.0096406)
Supplement: Figure S1a–e — Results of the associations between the smoking-alcohol subgroups and components of MetS, according to BMI class. Adjusted for age (centered at the mean age of the total population (45y)), sex and the number of medications used. * indicates a significant difference within each smoking subgroup relative to the reference group of non-drinkers (shaded shape); P value≤0.001. ** indicates a significant difference within each smoking subgroup relative to the reference group of non-drinkers (shaded shape); P value≤0.004. N: non-smokers; F: former smokers; C1: smokers of <20 g tobacco/day; C2: smokers of ≥20 g tobacco/day. 0: non-drinker; 1: ≤1 drink/day; 2: >1–2 drinks/day; 3: >2 drinks/day. BMI = body mass index; BG = fasting blood glucose; DBP = diastolic blood pressure; SBP = systolic blood pressure; TG = triglycerides; WC = waist circumference. (DOCX) [file pone.0096406.s001.docx]

**Supporting figures**

**Figure S1a-e. Results of the associations between the smoking-alcohol subgroups and components of MetS, according to BMI class.**

Adjusted for age (centered at the mean age of the total population (45y)), sex and the number of medications used.

* indicates a significant difference within each smoking subgroup relative to the reference group of non-drinkers (shaded shape); P value ≤ 0.001.

** indicates a significant difference within each smoking subgroup relative to the reference group of non-drinkers (shaded shape); P value ≤ 0.004.

N: non-smokers; F: former smokers; C1: smokers of <20 g tobacco/day; C2: smokers of ≥20 g tobacco/day.

0: non-drinker; 1: ≤1 drink/day; 2: >1-2 drinks/day; 3: >2 drinks/day.

BMI = body mass index; BG = fasting blood glucose; DBP = diastolic blood pressure; SBP = systolic blood pressure; TG = triglycerides; WC = waist circumference.

**Figure S1a

**

**Figure S1b**





**Figure S1c**





**

Figure S1d**

**Figure S1e**
