# Supplementary material for: Combined Effects of Smoking and Alcohol on Metabolic Syndrome: The LifeLines Cohort Study
Source: PLoS One. 2014 Apr 29;9(4):e96406. doi: 10.1371/journal.pone.0096406 (PMC4004580; doi:10.1371/journal.pone.0096406)
Supplement: Table S1 — Characteristics of the total study population by alcohol subgroup. (DOCX) [file pone.0096406.s002.docx]

**Table S1.** Characteristics of the total study population by alcohol subgroup.

| Characteristics | Non-drinker | ≤1 drink/day | >1 to 2 drinks/day | > 2 drinks/day | *P* value |
| --- | --- | --- | --- | --- | --- |
| n (%) | 10,499 (16.4) | 32,573 (50.9) | 14,378 (22.4) | 6,596 (10.3) |  |
| Age, yrs | 46 ± 12 | 44 ± 12 | 46 ± 12 | 45 ± 12 | ≤0.001 |
| Sex (m (%)/f) | 2,453 (23.4) /  8,046 | 12,636 (38.8) / 19,937 | 8,516 (59.2) /  5,862 | 5,119 (77.6) /  1,477 |  |
| BMI, kg/m^2^ | 26.8 ± 5.1 | 25.7 ± 4.1 | 25.7 ± 3.6 | 26.1 ± 3.7 | ≤0.001 |
| SBP, mmHg | 125/126 ± 16/17 | 125 ± 15/16 | 127/128 ± 15 | 131/132 ± 15 | ≤0.001 |
| DBP, mmHg | 73/74 ± 9 | 73 ± 9 | 75 ± 9 | 77 ± 10 | ≤0.001 |
| Total cholesterol, mmol/L | 5.0 ± 1.0 | 5.0 ± 1.0 | 5.1 ± 1.0 | 5.3 ± 1.0 | ≤0.001 |
| LDL-C, mmol/L | 3.18 ± 0.88 | 3.17 ± 0.89 | 3.28 ± 0.91 | 3.38 ± 0.93 | ≤0.001 |
| HDL-C, mmol/L | 1.43 ± 0.37 | 1.48 ± 0.39 | 1.50 ± 0.42 | 1.45 ± 0.40 | ≤0.001 |
| Triglycerides, mmol/L | 1.00 (0.70-1.38) | 0.97 (0.69-1.31) | 1.06 (0.74-1.44) | 1.22 (0.82-1.72) | ≤0.001 |
| Blood glucose, mmol/L | 4.95 (4.60-5.20) | 4.91 (4.60-5.20) | 4.99 (4.60-5.30) | 5.09 (4.70-5.40) | ≤0.001 |
| Waist circumference, cm | 91 ± 14 | 89 ± 12 | 91 ± 11 | 94 ± 11 | ≤0.001 |
| *Smoking status* |  |  |  |  |  |
| Non-smoker, n (%) | 6,300 (60.0) | 16,710 (51.3) | 4,926 (34.3) | 1,682 (25.5) | ≤0.001 |
| Former smoker, n (%) | 2,548 (24.3) | 10,291 (31.6) | 5,637 (39.2) | 2,317 (35.1) | ≤0.001 |
| <20 gram tobacco/day, n (%) | 1,247 (11.9) | 4,649 (14.3) | 3,148 (21.9) | 1,824 (27.7) | ≤0.001 |
| ≥20 gram tobacco/day, n (%) | 404 (3.8) | 923 (2.8) | 667 (4.6) | 773 (11.7) | ≤0.001 |
| *Medication use* |  |  |  |  |  |
| No medication, n (%) | 5,842 (55.6) | 20,948 (64.3) | 10,050 (69.9) | 4,590 (69.6) | ≤0.001 |
| ≤5 sorts of medication, n (%) | 4,242 (40.4) | 11,094 (34.1) | 4,150 (28.9) | 1,912 (29.0) | ≤0.001 |
| >5 sorts of medication, n (%) | 415 (4,0) | 531 (1.6) | 178 (1.2) | 94 (1.4) | ≤0.001 |
| BP-lowering medication, n (%) | 1,247 (11.9) | 2,368 (7.3) | 1,023 (7.1) | 549 (8.3) | ≤0.001 |
| Statin use, n (%) | 542 (5.2) | 1,067 (3.3) | 562 (3.9) | 306 (4.6) | ≤0.001 |
| TG-lowering medication, n (%) | 19 (0.2) | 19 (0.1) | 9 (0.1) | 7 (0.1) | ≤0.001 |
| Type 2 diabetes, n (%) | 231 (2.2) | 329 (1.0) | 120 (0.8) | 60 (0.9) | ≤0.001 |
| Oral anti-hyperglycaemic medication, n (%) | 172 (1.6) | 259 (0.8) | 95 (0.7) | 48 (0.7) | ≤0.001 |
| % fulfilling ≥ 3 out of 5 MetS criteria | 2,066 (19.7) | 4,249 (13.0) | 2,044 (14.2) | 1,313 (19.9) | ≤0.001 |

Table legend:

Data are presented as mean ± SD, or geometric mean (interquartile range).

Abbreviations: BMI = body mass index, SBP = systolic blood pressure, DBP = diastolic blood pressure, HDL-C = high density lipoprotein cholesterol, TG = triglycerides, BP = blood pressure, MetS = metabolic syndrome.
