# Supplementary material for: Combined Effects of Smoking and Alcohol on Metabolic Syndrome: The LifeLines Cohort Study
Source: PLoS One. 2014 Apr 29;9(4):e96406. doi: 10.1371/journal.pone.0096406 (PMC4004580; doi:10.1371/journal.pone.0096406)
Supplement: Table S2 — Distribution of the study population across the smoking and alcohol subgroups, according to BMI class. (DOCX) [file pone.0096406.s003.docx]

**Table S2.** Distribution of the study population across the smoking and alcohol subgroups, according to BMI class.

| BMI < 25 kg/m^2^ | Non-smoker (n=14,739) | | Former smoker (n=8,282) | | Moderate smoker (n=5,448) | | Heavy smoker (n=1,133) | |
| --- | --- | --- | --- | --- | --- | --- | --- | --- |
| All, n (%) | **MetS, n (%)** | **All, n (%)** | **MetS, n (%)** | **All, n (%)** | **MetS, n (%)** | **All, n (%)** | **MetS, n (%)** |  |
| Non-drinker | 2791 (18.9) | 61 (2.2) | 864 (10.4) | 41 (4.7) | 554 (10.2) | 25 (4.5) | 159 (14.0) | 13 (8.2) |
| ≤1 drink/day | 8823 (59.9) | 146 (1.7) | 4364 (52.7) | 110 (2.5) | 2373 (43.6) | 67 (2.8) | 373 (32.9) | 22 (5.9) |
| >1 to 2 drinks/day | 2426 (16.5) | 48 (2.0) | 2299 (27.8) | 62 (2.7) | 1636 (30.0) | 75 (4.6) | 293 (25.9) | 16 (5.5) |
| >2 drinks/day | 699 (4.7) | 19 (2.7) | 755 (9.1) | 32 (4.2) | 885 (16.2) | 33 (3.7) | 308 (27.2) | 22 (7.1) |

| BMI 25-30 kg/m^2^ | Non-smoker (n=10,952) | | Former smoker (n=9,179) | | Moderate smoker (n=5,157) | | Heavy smoker (n=1,149) | |
| --- | --- | --- | --- | --- | --- | --- | --- | --- |
| All, n (%) | **MetS, n (%)** | **All, n (%)** | **MetS, n (%)** | **All, n (%)** | **MetS, n (%)** | **All, n (%)** | **MetS, n (%)** |  |
| Non-drinker | 2190 (20.0) | 407 (18.6) | 1026 (11.2) | 225 (21.9) | 458 (11.0) | 119 (26.0) | 137 (11.9) | 44 (32.1) |
| ≤1 drink/day | 5980 (54.6) | 798 (13.3) | 4320 (47.1) | 762 (17.6) | 1737 (41.8) | 341 (19.6) | 383 (33.3) | 101 (26.4) |
| >1 to 2 drinks/day | 2025 (18.5) | 263 (13.0) | 2644 (28.8) | 465 (17.6) | 1218 (29.3) | 221 (18.1) | 277 (24.1) | 79 (28.5) |
| >2 drinks/day | 756 (6.9) | 111 (14.7) | 1189 (13.0) | 276 (23.2) | 744 (17.9) | 172 (23.1) | 352 (30.6) | 108 (30.7) |

| BMI ≥30 kg/m^2^ | Non-smoker (n=3,928) | | Former smoker (n=3,332) | | Moderate smoker (n=1,263) | | Heavy smoker (n=485) | |
| --- | --- | --- | --- | --- | --- | --- | --- | --- |
| All, n (%) | **MetS, n (%)** | **All, n (%)** | **MetS, n (%)** | **All, n (%)** | **MetS, n (%)** | **All, n (%)** | **MetS, n (%)** |  |
| Non-drinker | 1319 (33.6) | 601 (54.4) | 658 (19.7) | 331 (50.3) | 235 (18.6) | 128 (54.5) | 108 (22.3) | 71 (65.7) |
| ≤1 drink/day | 1907 (48.5) | 759 (39.8) | 1607 (48.2) | 764 (47.5) | 539 (42.7) | 279 (51.8) | 167 (34.4) | 100 (59.9) |
| >1 to 2 drinks/day | 475 (12.1) | 213 (44.8) | 694 (20.8) | 362 (52.2) | 294 (23.3) | 171 (58.2) | 97 (20.0) | 69 (71.1) |
| >2 drinks/day | 227 (5.8) | 119 (52.4) | 373 (11.2) | 217 (58.2) | 195 (15.4) | 127 (65.1) | 113 (23.3) | 77 (68.1) |

Abbreviations: BMI= body mass index, MetS= metabolic syndrome.
